# Translocation pause of remdesivir-containing primer/template RNA duplex within SARS-CoV-2’s RNA polymerase complexes

**DOI:** 10.3389/fmolb.2022.999291

**Published:** 2022-10-25

**Authors:** Yuanjun Shi, Jimin Wang, Victor S. Batista

**Affiliations:** ^1^ Department of Chemistry, Yale University, New Haven, CT, United States; ^2^ Department of Molecular Biophysics and Biochemistry, Yale University, New Haven, CT, United States

**Keywords:** RDV, remdesivir, primer extension pause, MD simulation, pyrophosphorylysis, translocation, RNA polymerase, two-metal ion catalysis

## Abstract

The mechanism of remdesivir incorporation into the RNA primer by the RNA-dependent RNA polymerase (RdRp) of severe acute respiratory syndrome coronavirus-2 (SARS-CoV-2) remains to be fully established at the molecular level. Here, we compare molecular dynamics (MD) simulations after incorporation of either remdesivir monophosphate (RMP) or adenosine monophosphate (AMP). We find that the Mg^2+^-pyrophosphate (PPi) binds more tightly to the polymerase when the added RMP is at the third primer position than in the AMP added complex. The increased affinity of Mg^2+^-PPi to the RMP-added primer/template (P/T) RNA duplex complex introduces a new hydrogen bond of a substituted cyano group in RMP with the K593 sidechain. The new interactions disrupt a switching mechanism of a hydrogen bond network that is essential for translocation of the P/T duplex product and for opening of a vacant NTP-binding site necessary for next primer extension. Furthermore, steric interactions between the sidechain of S861 and the 1′-cyano group of RMP at position *i*+3 hinders translocation of RMP to the *i* + 4 position, where *i* labels the insertion site. These findings are particularly valuable to guide the design of more effective inhibitors of SARS-CoV-2 RNA polymerase.

## Introduction

Remdesivir is an antiviral drug that inhibits viral replication after it is converted into the triphosphate form and bound to the active site of RNA-dependent RNA polymerase (RdRp) for its incorporation into the RNA primer (Beigel et al., 2020; Cohen and Kupferschmidt, 2020; Frediansyah et al., 2021; Gordon et al., 2020a,b; Yates and Seley-Radtke, 2019). However, its actual inhibition mechanism remains to be fully established at the molecular level. Here, we focus on the analysis of specific interactions at the active site of RdRp upon each incorporation of remdesivir monophosphate (RMP). These interactions provide key insights into how RMP can alter the polymerization reaction by affecting the translocation of the RNA duplex that is essential for primer extension during the nucleotide addition cycle. The results reported may help to guide the development of more potent and specific inhibitors of RdRp for the treatment of viral infections.

The replication-transcription complex (RTC) of SARS-CoV-2 is composed of RNA-dependent RNA polymerase (RdRp or nsp12), nsp7, nsp8, nsp13, and the template/primer (P/T) RNA duplex. RdRp is the enzyme that catalyzes the replication and transcription of viral RNAs essential for viral replication (Chen et al., 2020; Yan et al., 2020). It is targeted by remdesivir (RDV), a 1′-cyano adenosine analogue that binds to the active site of RdRp more strongly than adenosine. RDV is an analog of adenosine and incorporated into RNA by establishing complementary base pair interactions with uridine (Yates and Seley-Radtke, 2019). It was first shown to exhibit some efficacy against the *Ebola* virus and more recently against SARS-CoV-2 (Tchesnokov et al., 2019; Wang Y. et al., 2020). Compared to other nucleotide analogue mutagens (i.e., ribavirin and favilavir), RDV is more selective since it pairs only with uridine (Byléhn et al., 2021). Its modified 1′-C linkage between the ribosyl moiety and the base of RDV is designed to counterbalance a strong electron-withdrawing cyano substitution at the C1′ position and has a fully extendable 3′-OH (Yates and Seley-Radtke, 2019).

**FIGURE 1:**
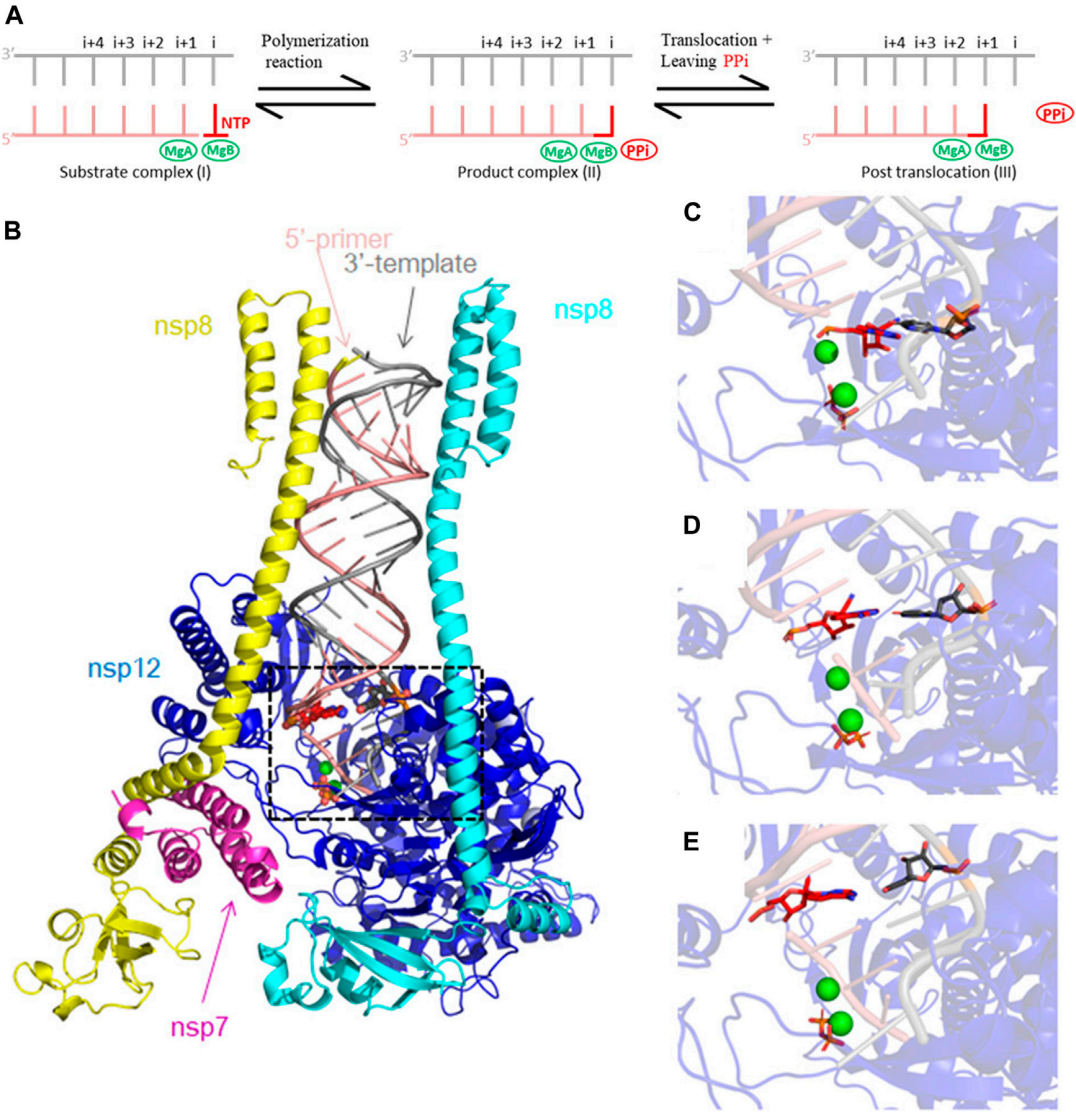
Cryo-EM RTC 6xez structure of SARS-CoV-2 used for our MD simulations. **(A)** Schematic diagram of RNA replication and translocation processes. The replication starts with NTP binding to position *i* (I). Phosphodiester bond is formed, and the nucleotide is incorporated into the 3′ end of the primer. PPi is cleaved from NTP (II). Upon release of PPi, the 3′ end of the primer translocates by one nucleotide (III). **(B)** Our MD simulation model includes the 6xez structure after removal of two nsp13 helicase subunits, consisting of subunits of nsp7 (magenta), nsp8 (yellow and cyan), nsp12 (blue) and the RNA template/primer duplex (pink and silver). Mg^2+^ ions are shown in green, and PPi is shown in orange and RMP is shown in red. **(C–E)** Close-up views of the modeled RMP at position *i* + 1, *i* + 2 and *i* + 3 used in this study.

The effect of RDV remains unclear because the primer with RMP added at positions *i*, *i* + 1, and *i* + 2 can be extended efficiently, where *i* labels the insertion site while *i* + 1 and *i* + 2 indicate sites after one- and two-base pair translocations of the RNA duplex, respectively (Figure 1A) (Gordon et al., 2020b). Further, SARS-CoV-2 RdRp exhibits a ∼3-fold higher selectivity for RMP over AMP at position *i* (Gordon et al., 2020b), an observation that has been used to rule out the chain termination hypothesis (Gordon et al., 2020a). Further, RDV is not necessarily a direct-acting inhibitor since it does not inhibit the RNA synthesis by RdRp of SARS-CoV-2. Most likely, RDV is an indirect-acting inhibitor for viral replication. RMP-containing RNA may not be functional for either translation (i.e., translational inhibition) or for the second pass of (+)-sense RNA synthesis (i.e., replicational inhibition) or both (Wang et al., 2021), the latter supported by experimental data (Tchesnokov et al., 2020). However, after incorporation of RMP, the RNA duplex accumulates with RMP at the third primer position under low concentration of incoming NTPs but not under physiological conditions (Gordon et al., 2020b; Yin et al., 2020). So, RMP slows down the primer extension significantly due to a translocation pause only at low NTP concentrations. This pause is completely eliminated with longer time intervals of the primer-extension assay or at higher NTP concentrations (i.e., at the physiological concentrations) (Gordon et al., 2020b). Thus, RMP is a delayed inhibitor of RNA synthesis, but not a chain terminator of any kind.

Cryo-EM structures have shown that the sidechain of S861 is next to the cyano group of RMP when RMP is at the fourth primer position (Wang Q. et al., 2020; Gordon et al., 2020b; Kokic et al., 2021). Juxtaposition of the S861 sidechain and the cyano group in these structures leads to the hypothesis that an extra Oγ atom in the S861 sidechain could block the translocation of the RNA duplex product with RMP at the primer position *i* + 3, hindering translocation to *i*+4. That mechanistic hypothesis is further supported by the S861A mutational data (Wang Q. et al., 2020). However, how the increased concentration of NTPs can overcome this pause remains elusive and is addressed in this study.

The concentration-dependent pause is an important dynamic property of the RdRp, which we address by using molecular dynamics (MD) simulations. RNA polymerases often use pauses as mechanisms of transcriptional regulation (Saba et al., 2019). Elemental pauses are often coupled to translocation, backtracking, and cleavage. Pauses often occur during mismatch extension, helping to backtrack any mismatched nucleotides for mismatch removal (Malone et al., 2021). In this specific case of RMP-containing P/T complexes, the binding affinity of the Mg^2+^ ion/pyrophosphate (PPi) complex controls the transcriptional pause, translocation, pyrophosphorylysis, and eventually, possible cleavage by exoribonuclease. A common efficient translocation mechanism of P/T duplex is often powered by the release of pyrophosphate (Yin and Steitz, 2014). This is a basis for this study. However, for T7 RNA polymerase in the presence of high concentrations of PPi (i.e., 0.5–3 *m*M, which is not physiologically relevant after shifting equilibrium of the polymerization-pyrophosphorylysis reaction) (Guo and Rousa, 2006) and in some polymerases such as *E. coli* RNA polymerase (Abbondanzieri et al., 2005), the release of PPi is uncoupled with the translocation step, i.e., it fails to drive the forward translocation. This uncoupled event has been studied computationally (Golosov et al., 2010; Da et al., 2015; Da et al., 2017). For other polymerases, ratcheting motion has been proposed during which two strands of RNA duplex are translocated asynchrotronically, one strand at a time, with transient deformation of base-pairing geometry between the primer and template strands (Silva et al., 2014; Shu and Gong, 2016; Wang M. et al., 2020). In many cases, the two mechanisms are highly coupled.

In this study, we started with the RTC model of 6xez (Chen et a., 2020), with 2 Mg^2+^ ions bound in the pol active site, MgA and MgB. We studied dynamic properties of the RTC using MD simulations shortly before and immediately after polymerization and/or translocation (but not the translocation process itself since that would require longer MD simulations than presented in this study). Our study differs from other recent similar MD simulations that studied different aspects of RNA synthesis (Romero et al., 2021; Zhang et al., 2021). Remero et al., focused on binding of RTP in both open and closed states of the polymerase, which couples the P/T translocation with the conformation NTP-binding site (Romero et al., 2021). Zhang et al. focused on the RMP’s effect on chain termination, which misinterpreted the existing biochemical literature as explained above, and on possible effect to the proofreading activity (Zhang et al., 2021) MgA binds the carboxylates next to O3′ of the primer-terminal nucleotide to activate the attacking 3-hydroxyl (Steitz and Steitz, 1993; Steitz, 1999). MgB binds carboxylates and the triphosphate moiety of NTP to stabilize the leaving PPi. If the leaving PPi cannot leave, the likelihood of pyrophosphorylysis increases, which could effectively inhibit polymerization (Wang and Konigsberg, 2022). We have carried out MD simulations with RMP at primer positions *i, i* + 1, *i* + 2, and *i* + 3 and analyzed the MD-derived electron density maps to see the effect of RMP at each primer position on releasing of PPi. Our results show that the MgB-bound PPi is the most stable in the complex after RMP is translocated to primer position *i* + 3 when compared to those without RMP or those with RMP at other positions, forming multiple hydrogen bonds with R553, R555 and K621, and thereby preventing PPi release.

## Computational methods

The cryo-EM RTC structure of SARS-CoV-2 (6xez) was used as a starting point for our MD simulations after removing nsp13 helicase (Chen et al., 2020). Two Mg^2+^ ions (MgA and MgB) and PPi were added to the model. In addition, two Zn^2+^ ions were included in the structure as part of the Zn-Cys motif important for the stability of the nsp12. One reference set of MD simulations was carried out with three AMPs at position *i* + 1, *i* + 2, and *i* + 3 of the RNA duplex, followed up by three sets of MD simulations with one RMP replaced at each of the three positions (Figure 1). MD simulations of RMP/PPi and RTP at position *i* will be described elsewhere. Each set was run with two replicas. Schrödinger Maestro (Schrödinger, 2022) was used to prepare the complex structures. Protein Preparation Wizard of the suite was used to assign bond orders and protonation states and add missing side chains and hydrogens. These model complexes were placed in water boxes with a 15 Å cushion for the complex and Na^+^ ions were added to neutralize the systems. The parameter/topology files were created by the LEaP program from the AmberTools package (Cornell et al., 1995). MD simulations were run using NAMD (Kalé et al., 1999; Phillips et al., 2005). The system was equilibrated at 310 K in three steps before production run: the equilibration minimization of 1) solvent, 2) solvent and side chains, and 3) the whole system.

The MD simulation systems contained 21,695 atoms before addition of water molecules and counterions, and ∼276,852 atoms afterwards. About 70 Na^+^ ions were added as counterions to neutralize the whole system. The remdesivir parameters were generated by optimizing the molecule with B3LYP/6-31G* first, and then calculating the electrostatic potential for partial atomic charges (Gaussian09; Frisch et al., 2016). RESP calculation was run by antechamber to atomic partial charges whereas atomic charges of the nucleobase were kept to the same as those of ATP (Bayly et al., 1993). The 2 Mg^2+^ ions and PPi were parameterized using MCPB program in AmberTools, which can be used for building bonding interactions for ligand-binding metalloproteins, and which used Gaussian09 to idealize the geometry and to calculate the force constants and electrostatic potentials (Cornell et al., 1995; Meagher et al., 2003; Li and Merz, 2016). NPT ensemble was used. RMSD analysis was carried out for 100-ns MD trajectories.

For a 100-ns MD simulation production run, a 2-fs time step was used. The electron density (ED) maps derived from MD trajectories were calculated for the complex structure using CCP4 as previously described (Wang et al., 2022a; Wang et al., 2022b; Wang et al., 2022c; Winn et al., 2011), from which equilibrium structures were derived by fitting into and refining against MD-derived ED maps. To determine the consequences of RMP substitution in each position relative to AMP, ED map differences were calculated between the RMP- and AMP-bound complexes of MD trajectories, using the CCP4 suite (Winn et al., 2011). VMD was used to analyze the distribution of both the PPi positions (RMSD trajectory tool) and hydrogen-bond interactions (Hydrogen Bonds tool) (Humphrey et al., 1996). Equilibrated structures were manually fitted into MD-derived-ED maps using Coot (Emsley and Cowtan, 2004). All figures were made from fitted equilibrated structures and were visualized by PyMOL (Delano, 2022).

## Results

**FIGURE 2:**
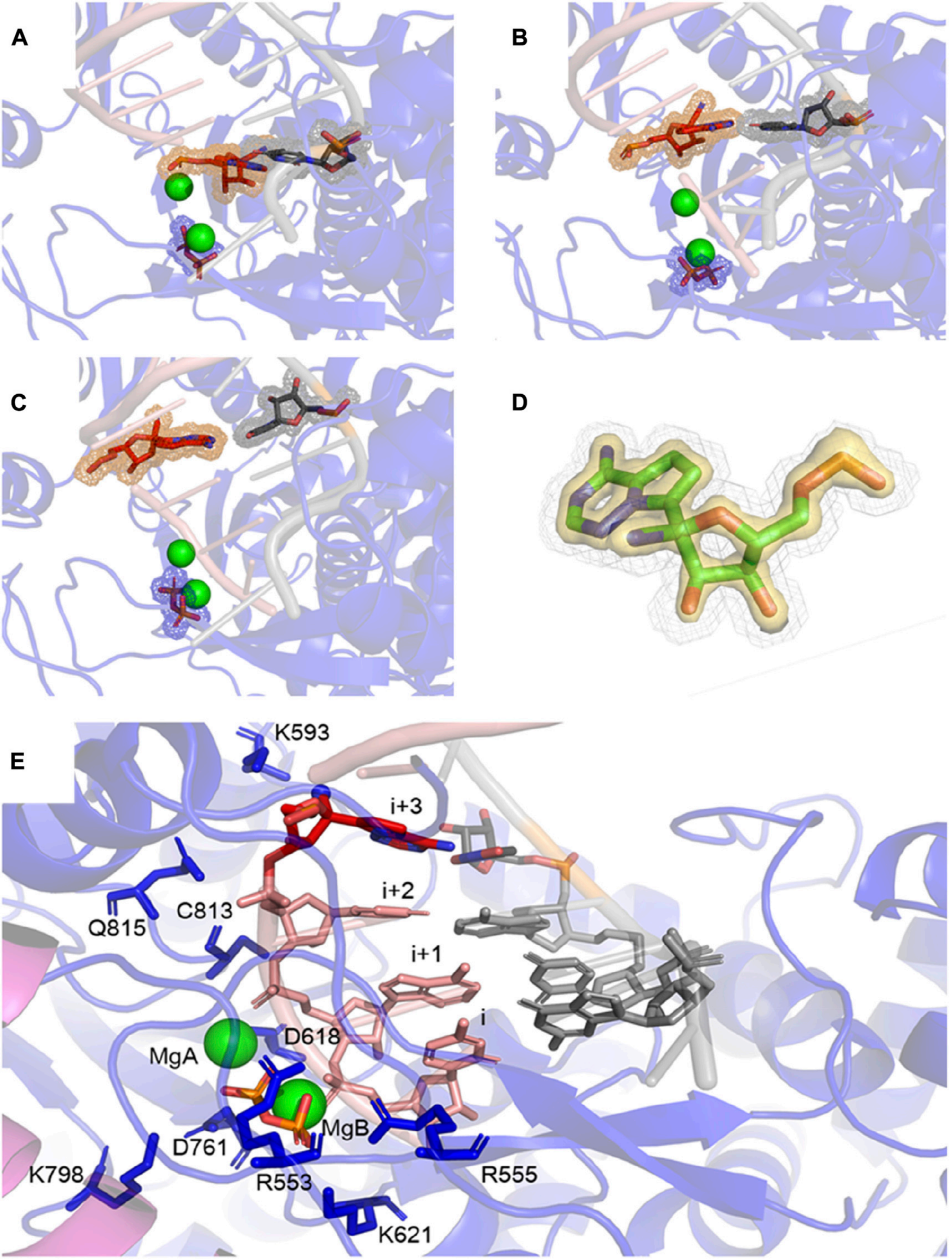
MD-derived ED maps. **(A–C)** MD-derived ED maps carved for the PPi and the (*i* + 1), (*i* + 2), and (*i* + 3) base pairs in the context of RNA duplexes. The electron densities for PPi, RMP and U which form Watson-Crick interactions are shown in mesh. **(D)** Close-up view of the MD-derived ED maps carved for the RMP in two contour levels (low contour level in silver mesh and high contour level in gold surface). **(E)** Close-up view of RMP at *i*+3 complex. PPi, (*i* + 1), (*i* + 2), and (*i* + 3) base pairs and surrounding residues are shown in sticks. Mg^2+^ ions are shown in spheres.

**FIGURE 3:**
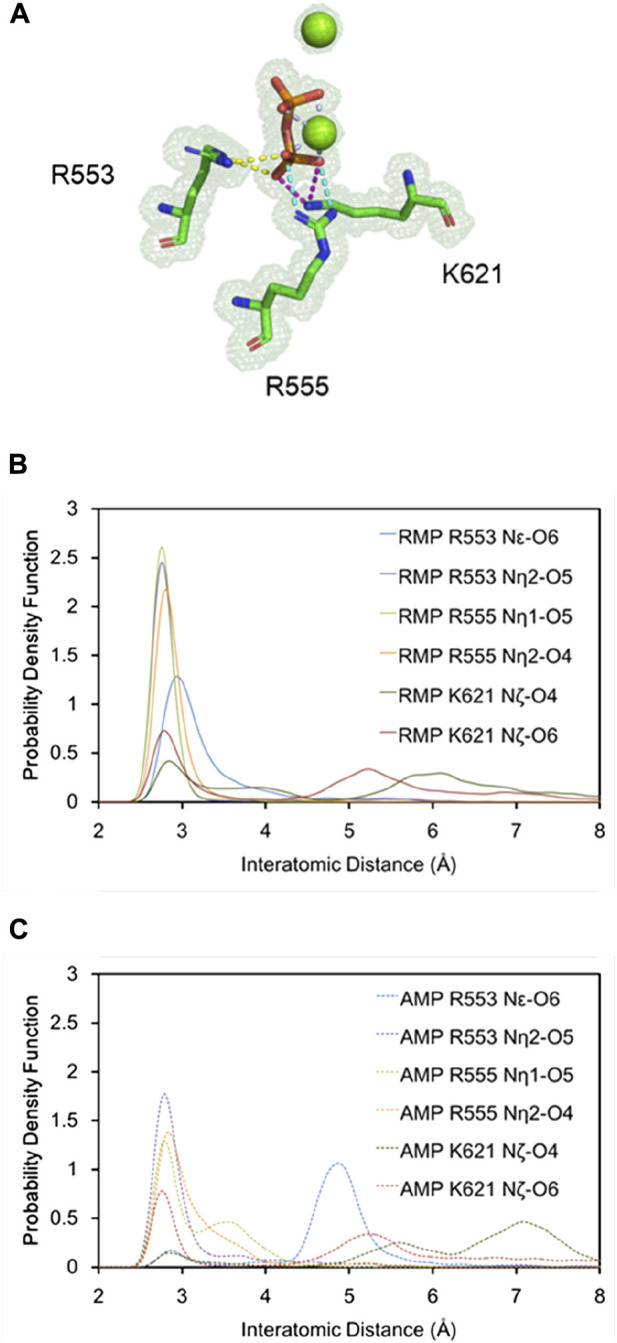
Detailed H-bonding interactions for PPi. **(A)** A close-up view of the MD-derived ED maps for PPi and interacting residues. **(B,C)** PDFs of the H-bond distance distribution for PPi and interacting residues with RMP (**B**, solid curves) and AMP (**C**, dashed curves) at position *i* + 3.

Figure 2 shows that RMP forms stable base pairs at all three positions *i* + 1, *i* + 2, and *i* + 3. PPi interacts with the Mg^2+^ ions mainly through electrostatic interactions in all three structures, although the strength of the interactions of PPi varies in the three complexes. The H-bond is stronger when RMP is in position *i* + 3 (the *i* + 3 complex). The H-bond interaction analysis shows that the PPi forms H-bonds with three surrounding amino acid residues R553, R555, and K621 (Figure 3), forming on average 1.52, 1.74 and 2.6 H-bonds per residue throughout the MD trajectories, respectively. For comparison, the average number of H-bonds with those residues in complex *i* + 1 is reduced to 1.09, 1.35, and 2.05, respectively, and for complex *i* + 2 to 1.24, 0.89, and 0.31, respectively. Moreover, the H-bond probability density functions (PDFs) for the PPi to each of its interaction partners are sharp with peaks around 3 Å when RMP is at position *i* + 3 whereas for AMP at position *i* + 3, the corresponding distributions are broader with some peak positions shifted by more than 2.0 Å (Figure 3). Therefore, multiple H-bonding interactions between the PPi and these residues (R553, R555, and K621) are primarily responsible for stabilizing the bound product PPi.

**FIGURE 4:**
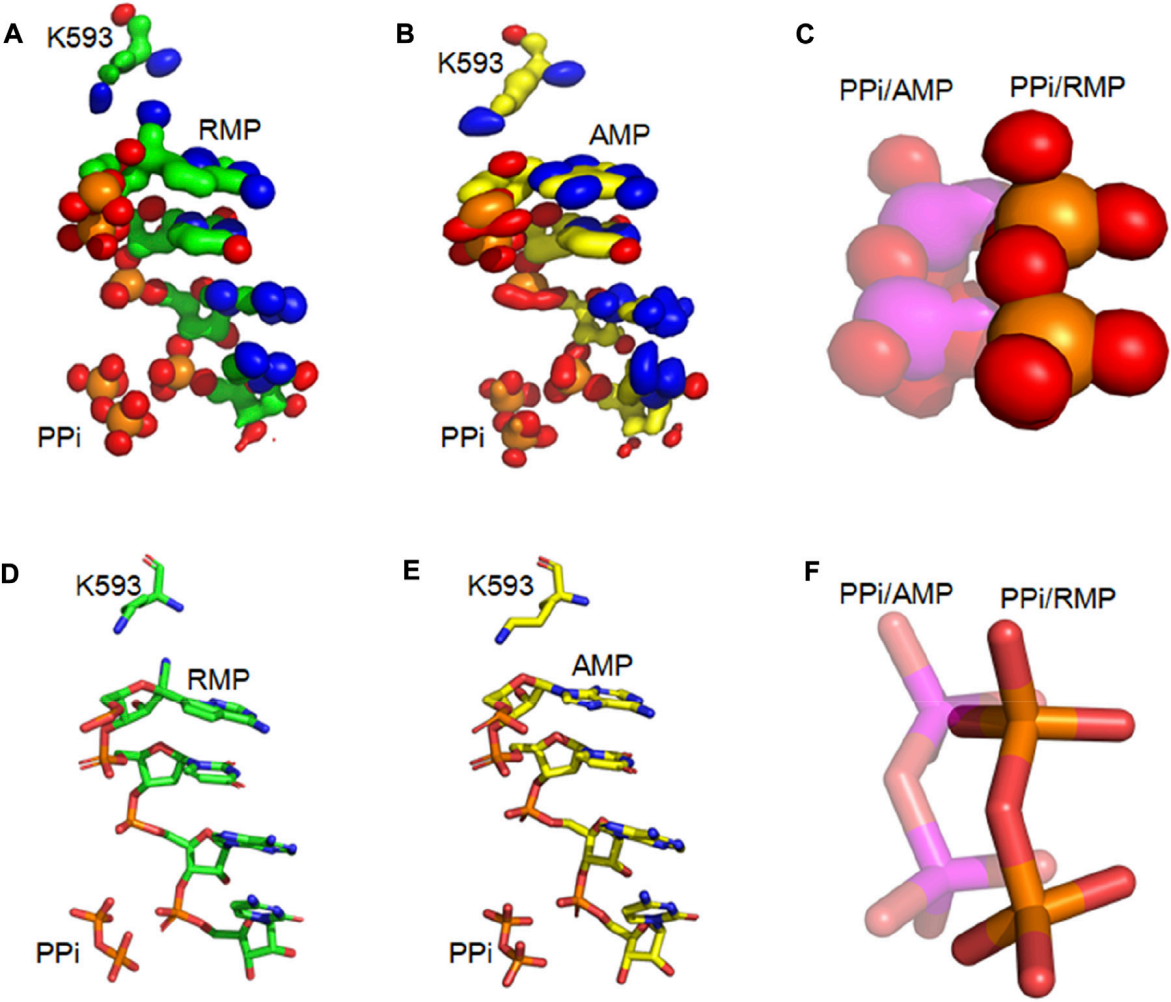
Iso-PDF Distribution of PPi and primer RNA strands **(A,B)** Iso-PDF for bases from the first three primer nucleotides, K593, and PPi for RMP **(A)** and AMP **(B)** at position *i* + 3. **(C)** Close-up view of iso-PDF for PPi (RMP structure: solid; and AMP structure: transparent). **(D)** The *i* + 3 RMP complex structure for showing K593, PPi, and 4 nucleotides of the primer strand. **(E)** Corresponding *i* + 3 AMP complex structure. **(F)** Superposition of the two complexes.

Our analysis of root-mean-square fluctuations (RMSF) from the average structure shows that the RMSF value for the PPi is 0.7 (0.3) Å and 1.3 (0.4) Å (numbers in parenthesis is uncertainty or one standard deviation of RMSF) in the *i* + 3 RMP and AMP complexes, respectively. These values are indicative of an increased stability of RMP relative to AMP at the *i* + 3 position since the more stable the binding is, the smaller the RMSF value (e.g., binding to a shallow well corresponds to large RMSF). Results of this analysis are consistent with the calculated iso-probability densities (Figure 4), showing that the probability isosurface for the PPi in the AMP structure is more smeared out (i.e., less stable configuration) than the PPi in the RMP structure.

For the *i* + 3 RMP complex, the RMSF analysis shows that the RMSFs for the three nucleotides of the primer strand at positions *i* to *i* + 2 are 0.8 (0.3) Å, 0.7 (0.2) Å, and 0.7 (0.2) Å, respectively. In contrast, the corresponding values for AMP complexes are 1.0 (0.3) Å, 1.2 (0.3) Å, and 0.8 (0.2) Å. In addition, the RMSFs for single RMP nucleotides at positions *i* + 1 to *i* + 3 are 0.8 (0.2) Å, 0.8 (0.3) Å and 0.8 (0.3) Å. In contrast, the corresponding values for corresponding single AMP nucleotides increase to 1.2 (0.3) Å, 1.0 (0.4) Å and 0.9 (0.3) Å. Clearly, the incorporated RMP increases the rigidity of the RNA duplex. Again, these results are consistent with the iso-probability density analysis. Therefore, incorporation of RMP stabilizes the nucleic duplex relative to the system without RMP.

**FIGURE5:**
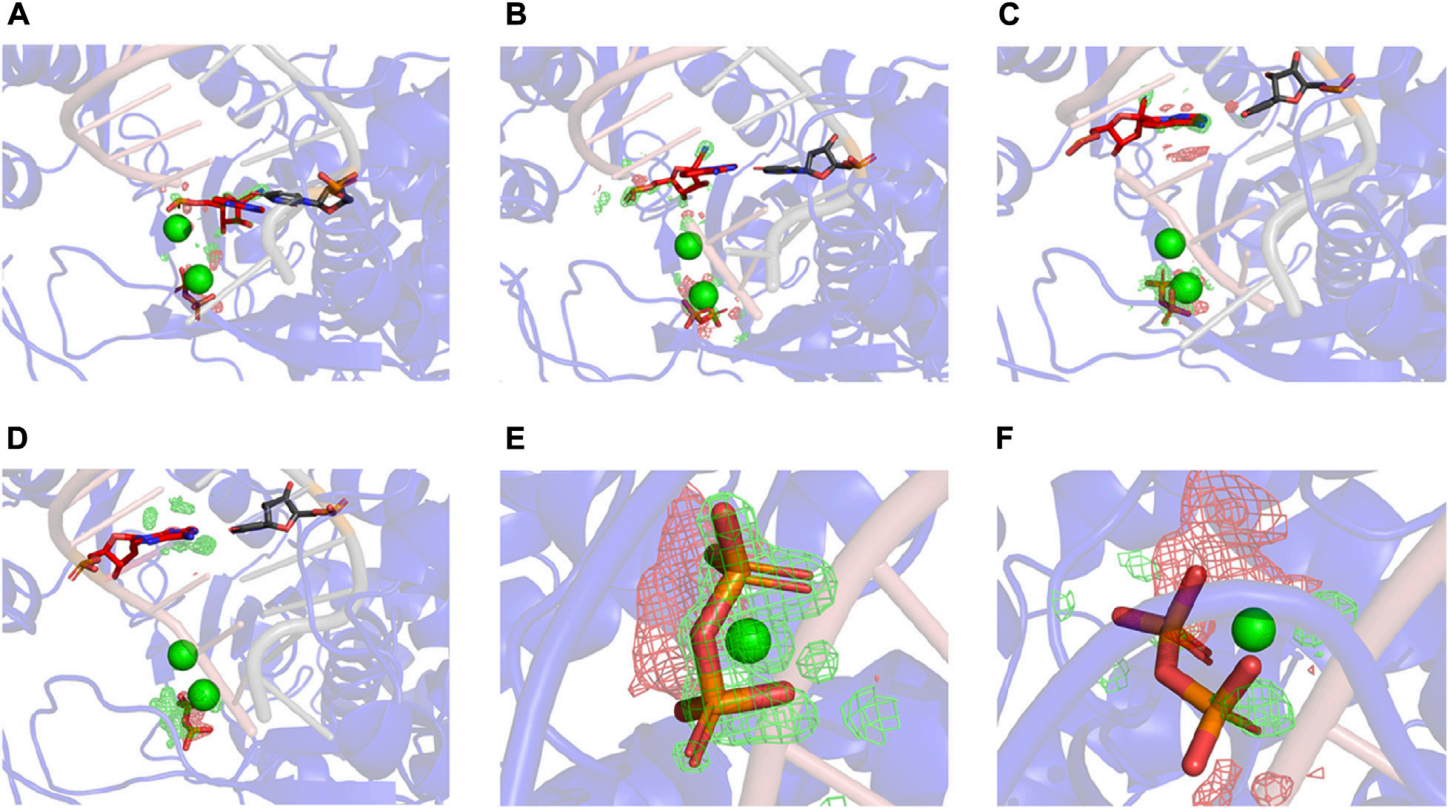
Differences between RMP and AMP complexes determined using difference ED maps. **(A–C)** Difference ED maps between RMP and AMP for the three paired structures. RMP complexes are aligned to the difference map. Green mesh indicates positive and red mesh indicates negative (contoured at ± 15σ). **(D)** *I*+3 AMP complex aligned to the difference map. **(E,F)** Close-up views of PPi of *i* + 3 and *i* + 2 complex.

For the *i* + 3 complexes, the difference MD-derived ED maps show that the largest difference in the entire map is observed on the PPi (Figure 5), and the second largest is on the cyano group of RMP. The largest difference on PPi is associated with both its change in position and *B*-factor when comparing the two complexes (i.e., PPi becomes much more ordered in the RMP complex since it gets displaced into a new position where it establishes better H-bonding interactions than in the AMP complex). For the *i* + 1 and *i* + 2 complexes, the larger differences are on the cyano group, while differences near the PPi are relatively very small (i.e., there is no difference in binding affinity of PPi between those pairs of complexes). All these observations support our hypothesis that the PPi binds more tightly when RMP is added to the primer strand and occupies position *i* + 3, which is consistent with the paused translocation.

**FIGURE 6:**
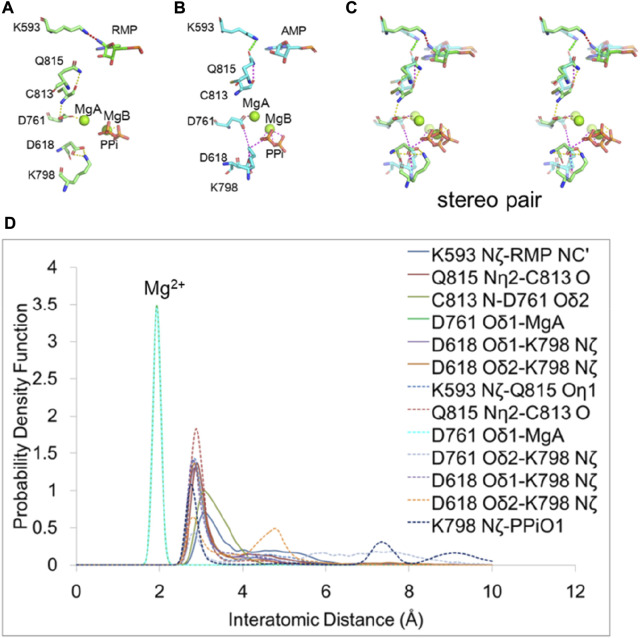
H-bonding network for allostery communication between the pol active site and the remote positions of RNA duplex. **(A,B)** Residues affected by RMP and AMP at position *i* + 3. H-bond interactions are shown in dash lines. The H-bond between K593 and RMP is shown in red and others are shown in yellow. The H-bond between K593 and Q815 is shown in green and others are shown in magenta. **(C)** Overlap of RMP (solid curves) and AMP (dashed curves) (transparent) structures in stereodiagram. **(D)** PDFs of the H-bond distance distribution for interacting residues with RMP and AMP at position *i* + 3. Peaks that are approximately at 2 Å are for interactions between Mg^2+^and amino acid residues. Others are for interactions between amino acid residues.

We analyzed the H-bond network between the RMP at primer position *i* + 3 and the PPi bound at a distance of over 15 Å apart to determine how the RMP at *i* + 3 stabilizes the PPi (Figure 6). We found that K593 adopted two different conformations when comparing the two *i* + 3 complexes of RMP and AMP, potentially functioning as a switch for RNA duplex translocation. For the RMP complex, K593 interacts with either the cyano group of the RMP or the Q815 sidechain of the RdRp in a distributive manner but does not interact with both residues simultaneously. Since the cyano group of RMP interacts with K593, there is less interaction between K593 and Q815, so the sidechain of Q815 is displaced towards the backbone carbonyl of C813 and the backbone amide of C813 is displaced towards the sidechain of D761.

In the AMP complex (i.e., in the absence of an equivalent cyano group), K593 interacts solely with the sidechain of Q815 which in turn interacts along a H-bonding network with the backbone carbonyl group of C813. These interactions lead to a reduction of H-bonding strength between the backbone amide of C813 and the sidechain of D761, so D761 coordinates more strongly with MgB, weakening the MgB-PPi interaction. The carboxyl group of D761 repels that of D618 and changes its position. As a result, D618 interacts less with K798 and in turn K798 interacts more strongly with PPi, pulling PPi away from three other positively charged residues (R553, R555 and K621 that provide greater stabilization of the MgB-bound PPi in the RMP complex). Therefore, we find that weakening the interactions of PPi with R553, R555, and K621 after switching PPi to a new interaction with K798 may be essential for releasing PPi in this polymerase. This step is equivalent to the reopening of the Fingers domain of RB69 DNA polymerase (Franklin et al., 2001).

## Discussion

The mechanism for stalling primer extension by RMP has been suggested to be associated with a free energy barrier for translocation (Bravo et al., 2021; Kokic et al., 2021). However, the molecular origin of the proposed barrier remains unknown. Here, we find that when RMP is located at position *i* + 3, the RdRp complex remains in the pre-translocated state, which lacks the vacant NTP-binding site observed in the post-translocation state. Therefore, it prevents binding of the next incoming NTP. When RMP is replaced by AMP, the RdRp complex rapidly advances to the post-translocation state and the NTP binding site becomes vacant (Kokic et al., 2021). Stalling has been attributed to a physical barrier between the 1′-cyano group of RMP and the sidechain of S861 (Bravo et al., 2021), which has been confirmed in our MD simulations. There would be a steric clash between S861 and the cyano group of the added RMP if RdRp were translocated to position *i* + 4. Therefore, mutation of S861 reduces the stalling effect (Wang Q. et al., 2020). In addition, we find that even before translocation, RMP forms a H-bond with K593, which disrupts the interactions of PPi with K798 in a cascade of events within a H-bond network. The disrupted interactions enhance the ability of RMP remaining at position *i* + 3 to stall translocation.

## Concluding remarks

RMP acts as a delayed inhibitor and slows down the primer extension. During the first pass of viral RNA synthesis by the SARS-CoV-2 RNA-dependent RNA polymerase. When RMP is translocated to position *i* + 3, it induces a transcriptional pause under the reduced NTP concentrations. Our computational analysis points to a tighter binding of PPi as the physical basis of the pause. When RMP is in position *i* + 3, the cyano group of RMP forms hydrogen bonds with K593 and prevents a switch of a H-bonding network established by protein residues that are essential for primer/template translocation. This increases the binding stability of the PPi product so that PPi blocks the translocation of RNA duplex and slows primer extension.

## Data Availability

The raw data supporting the conclusion of this article will be available at http://ursula.chem.yale.edu/~batista/pubs/index.html
